# Recombinant Human Thymosin Beta-4 Protects against Mouse Coronavirus Infection

**DOI:** 10.1155/2021/9979032

**Published:** 2021-04-21

**Authors:** Rui Yu, Yunyun Mao, Kai Li, Yanfang Zhai, Yue Zhang, Shuling Liu, Yuemei Gao, Zhengshan Chen, Yanhong Liu, Ting Fang, Mengsu Zhao, Ruihua Li, Junjie Xu, Wei Chen

**Affiliations:** Beijing Institute of Biotechnology, Beijing 100071, China

## Abstract

Coronaviruses (CoVs) are enveloped and harbor an unusually large (30–32 kb) positive-strand linear RNA genome. Highly pathogenic coronaviruses cause severe acute respiratory syndrome (SARS) (SARS-CoV and SARS-CoV-2) and Middle East respiratory syndrome (MERS) (MERS-CoV) in humans. The coronavirus mouse hepatitis virus (MHV) infects mice and serves as an ideal model of viral pathogenesis, mainly because experiments can be conducted using animal-biosafety level-2 (A-BSL2) containment. Human thymosin beta-4 (T*β*4), a 43-residue peptide with an acetylated N-terminus, is widely expressed in human tissues. T*β*4 regulates actin polymerization and functions as an anti-inflammatory molecule and an antioxidant as well as a promoter of wound healing and angiogenesis. These activities led us to test whether T*β*4 serves to treat coronavirus infections of humans. To test this possibility, here, we established a BALB/c mouse model of coronavirus infection using mouse CoV MHV-A59 to evaluate the potential protective effect of recombinant human T*β*4 (rhT*β*4). Such a system can be employed under A-BSL2 containment instead of A-BSL3 that is required to study coronaviruses infectious for humans. We found that rhT*β*4 significantly increased the survival rate of mice infected with MHV-A59 through inhibiting virus replication, balancing the host's immune response, alleviating pathological damage, and promoting repair of the liver. These results will serve as the basis for further application of rhT*β*4 to the treatment of human CoV diseases such as COVID-19.

## 1. Introduction

Coronaviruses (CoVs) that cause severe acute respiratory syndrome (SARS), such as SARS-CoV/SARS-CoV-2, and Middle East respiratory syndrome (MERS), such as MERS-CoV, are members of a large family of enveloped, single-stranded, positive-strand RNA viruses. Coronaviruses infect vertebrates and may cause acute respiratory distress syndrome (ARDS) with high morbidity and mortality rates, depending on species [1, 2].

The novel coronavirus pneumonia (COVID-19) outbreak and subsequent global pandemic caused by SARS-CoV-2 commenced around the end of 2019. When this manuscript was submitted for publication, there were more than 100 million cases and more than 2 million deaths worldwide. SARS-CoV-2, which is the seventh known coronavirus that infects humans, enters the respiratory system through the upper respiratory tract and eventually infects alveolar cells [3]. The virus continuously proliferates, destroys the alveoli, and enters the capillaries. As blood circulates, the “battlefield” is further expanded, causing pathologies such as conjunctivitis, pneumonia, diarrhea, anosmia, abnormal liver function, renal failure, and heart damage that culminates in severe disease [4].

Mouse coronaviruses, also known as mouse hepatitis viruses (MHVs), are histologically classified as respiratory strains such as MHV-1, MHV-2, MHV-3, MHV-A59, and MHV-JHM as well as enterophilic strains such as MHV-y and MHV-R1. The respiratory strain MHV-A59 used here, as well as SARS-CoV and SARS-CoV-2, are *β*-coronaviruses. MHV-A59 can spread to numerous target organs after replicating in nasal epithelial cells. MHV is an excellent model for studying the pathogenesis, tropism, and virulence, as well as the host's antiviral immune response. Further, MHV serves as a model for SARS-CoV infection. Moreover, MHV readily infects mice but not humans, and experiments can be conducted using animal-biosafety level-2 (A-BSL2) containment instead of A-BSL3 that is required for handling SARS-CoV and SARS-CoV-2 [5].

Thymosin beta-4 (T*β*4), an N-acetylated 43-amino acid residue peptide (molecular mass, 4960.5 Da; isoelectric point, 4.6) [6], is widely distributed among tissues of mammals and other vertebrates [7]. T*β*4 possesses numerous biological activities such as inhibiting inflammation [8–13], preventing infection of wounds [14], inhibiting apoptosis [15–17] and scar formation [9, 15, 18], promoting cell migration and angiogenesis [19–21], accelerating collagen deposition, and upregulating the production of laminin-5 [22–27]. Nonclinical and clinical studies show the clinical importance of T*β*4 for treating dry eye, cardiac failure [19, 28], lung fibrosis [29, 30], septic shock [8], liver injury [31], and acute ischemic kidney injury [32, 33]. T*β*4 facilitates cardioprotection and regeneration of the ischemic heart by promoting the survival of cardiomyocytes through modulating the inflammatory environment and promoting neovascularization [28]. T*β*4 possesses antioxidant, anti-inflammatory, and antifibrotic potential as demonstrated by studies of a mouse model of chronic liver injury [31]. In a rat model of ischemic acute kidney injury, the beneficial therapeutic effects of T*β*4 are mediated through inhibition of extracellular matrix remodeling and apoptosis, as well as modulation of renal redox status and inflammation [33].

The multiple activities of T*β*4 may be useful for treating coronavirus infection. Specifically, T*β*4 may exert its biological activities at the molecular, cellular, and tissue levels to inhibit the “cytokine storm” caused by coronaviruses. Further, T*β*4 may play an important role in reconstituting the immune response, inhibiting inflammation, reducing lung injury, ameliorating pulmonary fibrosis, and reducing systemic sequelae of coronavirus pneumonia.

As proof of principle, here, we used recombinant human T*β*4 (rhT*β*4) to evaluate the protective effect of rhT*β*4 in a mouse model of MHV-A59 infection. Compared with chemically synthesized T*β*4, rhT*β*4 has numerous advantages such as uniform quality, unlimited scale of production, relatively low production costs, and minimal adverse environmental effects. The results provide an experimental basis for applying rhT*β*4 to the treatment of human CoV infection.

## 2. Materials and Methods

### 2.1. Virus, Cells, Mice, and rhT*β*4

MHV-A59 was acquired from Guangdong Laboratory Animals Monitoring Institute of China. DBT cells were provided by the Chinese Experimental Animal Resources Research Institute for Food and Drug Control. Uninfected and MHV-A59-infected cells were passaged and preserved in our laboratory. Female SPF BALB/c mice (18–20 g) were purchased from Beijing Vital River Laboratory Animal Technology Co., Ltd. All animal experiments were performed according to protocols approved by the Institutional Animal Care and Use Committee of Beijing Institution of Biotechnology (Identification code: IACUC-DWZX-2020-041; date of approval: 30 April 2020). Virus production and all animal experiments were performed in an A-BSL2 laboratory, and rhT*β*4 was expressed and purified using our patented technology (Chinese invention patent No: ZL200910135972.0 and ZL201910498316.0) [34].

### 2.2. Reagents and Kits

The culture medium DMEM and fetal bovine serum (FBS) are products of Gibco. A Bio-Plex Pro Mouse Cytokine 23-plex Assay Kit (M60009RDPD) was purchased from Bio-Rad Laboratories, Inc. A Mouse C-Reactive Protein ELISA Kit (ab222511) was purchased from Abcam. An MHV-Ab ELISA Kit (DG30747M) was purchased from Beijing Winter Song Boye Biotechnology Co. Ltd, China. An RNeasy Plus Mini Kit (74136), a QuantiTect Reverse Transcription Kit (205311), and a QuantiTect Probe PCR Kit (204343) were purchased from QIAGEN. Paraformaldehyde (4%) was purchased from Beijing Solarbio Technology Co. Ltd, China. RNAstore reagent was purchased from TIANGEN Biotech (Beijing) Co. Ltd, China. The preparation of sections for histopathological analysis and hematoxylin-eosin (HE) staining of mouse organs was performed by Beijing Soonbio Technology Co., Ltd, China.

### 2.3. Virus Propagation and Titer Determination

DBT cells were cultured at 37°C in an atmosphere containing 5% CO_2_ in DMEM containing 5% FBS in culture flasks filled to 90% capacity. The virus growth medium was then replaced with DMEM containing 2% FBS, and MHV-A59 (multiplicity of infection = 10) was added. The cells were monitored each day. When >75% of the cells exhibited cytopathic effects, the culture flask was frozen at –80°C and thawed. The supernatant was collected and centrifuged for 5 min at 1000 × g to remove cells. The supernatants were frozen and stored at –80°C. The TCID_50_ of the virus was calculated using the method of Reed-Muench [35].

### 2.4. Infection of Mice and Administration of rhT*β*4

**Figure 1:**
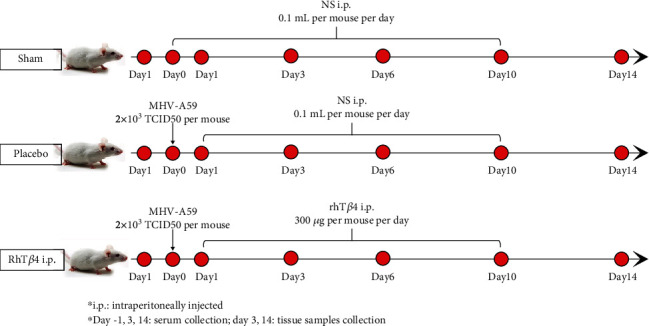
Process of mice experiment. Each of the mice in the sham group was intraperitoneally injected with 0.1 mL of normal saline (NS) daily from days 0 to 10. The mice in the placebo group were each intraperitoneally injected with 2 × 10^3^ TCID_50_ of MHV-A59 on day 0, and 0.1 mL NS was intraperitoneally injected into each mouse once daily from days 1 to 10. The mice in the rhT*β*4 group were intraperitoneally injected with 2 × 10^3^ TCID_50_ of MHV-A59 per mouse on day 0, and 300 *μ*g of rhT*β*4 in 0.1 mL NS was intraperitoneally injected per mouse daily from days 1 to 10. The mice in each group were observed until day 14.

SPF BALB/mice (*n* = 24) were randomly divided into the groups as follows: sham, placebo, and rhT*β*4. Each of the eight mice in the sham group was intraperitoneally injected with 0.1 mL of normal saline (NS) daily from days 0 to 10 (Figure 1). The eight mice in the placebo group were each intraperitoneally injected with 2 × 10^3^ TCID_50_ of MHV-A59 on day 0, and 0.1 mL NS was intraperitoneally injected into each mouse once daily from days 1 to 10. The mice in the rhT*β*4 group were intraperitoneally injected with 2 × 10^3^ TCID_50_ of MHV-A59 per mouse on day 0, and 300 *μ*g of rhT*β*4 in 0.1 mL NS was intraperitoneally injected per mouse daily from days 1 to 10. The mice in each group were observed until day 14.

These experiments were then repeated with the same groups and dosing schedule, and the number of mice was increased to 12 in each group for next analysis.

The surviving mice were weighed on days 0, 2, 5, 8, and 14. Food consumption of each group was monitored by weighing the food on days 3, 6, 10, and 14. Blood was collected from the tail vein of surviving mice, and the serum was separated on days 1, 3, and 14. On days 3 and 14, 3 mice in each group were killed, and the liver, lung, and kidney tissues were collected. The tissues were stored in 4% paraformaldehyde or in RNAstore reagent. Two mice in the placebo group survived, and their data on day 14 were combined.

### 2.5. Analysis of Anti-MHV Antibodies

The tail vein blood of the surviving mice in each group was collected on day 14 into serum separator tubes. The blood was allowed to clot for 2 h at room temperature before centrifugation for 20 min at approximately 1000 × g. The freshly prepared serum was immediately assayed using the MHV-Ab ELISA Kit. A standard curve was generated using six concentrations of the standard, and the concentration of the anti-MHV antibodies was evaluated.

### 2.6. Quantitative Real-Time Polymerase Chain Reaction (qPCR) Analysis of Viral RNA in Mouse Tissues

The qPCR assays employed a TaqMan probe to quantify MHV-A59 RNA in liver, lung, and kidney tissues of mice 3 and 14 days postinfection. The sequences of the primers and TaqMan probe were designed according to the MHV-A59 sequence (GenBank No. FJ6742245) as follows: MHV-F: 5 ′–ggaacttctcgttgggcattatact–3′; MHV-R: 5′–tatgttgtgaaaatgataatcttgtggt–3′; MHV-probe: FAM-acatgctacggctcgtgtaaccgaactgt-BHQ1. A 130-bp DNA fragment which contained the T7 promoter sequence and a specific sequence from MHV-A59, was cloned into the pCloneEZ-TOPO-NRS-Amp vector (Taihe Biotechnology, Beijing, China) as a positive control. The copy numbers of the plasmid containing the 130 bp insert were estimated to serve as standards.

Tissue RNAs prepared using a RNeasy Plus Mini Kit were reverse transcribed with a QuantiTect Reverse Transcription Kit and quantitated according to the protocol supplied with the QuantiTect Probe PCR Kit. TaqMan reaction mixtures (20 *μ*L) contained 10 *μ*L of 2× Master Mix, 400 nM MHV-F or MHV-R primer, 200 nM MHV probe, and 2 *μ*L of cDNA template. After initial denaturation at 95°C for 10 min, 40 amplification cycles were performed, which included melting and annealing steps at 95°C, 15 s and 60°C and 60°s, respectively. Amplification, detection, and data analysis were performed using an ABI 7500 Real-Time PCR system (Applied Biosystems, USA).

### 2.7. Cytokine Assays

Cytokines in mouse sera were detected using a Bio-Plex Pro Mouse Cytokine 23-plex Assay Kit according to the protocol provided by the manufacturer. Data were acquired using a Bio-Plex 200 System (Bio-Rad, USA). The cytokines tested were as follows: IL-1*α*, IL-1*β*, IL-2, IL-3, IL-4, IL-5, IL-6, IL-9, IL-10, IL-12 p40, IL-12 p70, IL-13, IL-17A, eotaxin, G-CSF, GM-CSF, IFN-*γ*, KC, MCP-1 (MCAF), MIP-1*α*, MIP-1*β*, RANTES, and TNF-*α*.

### 2.8. C-Reactive Protein (CRP) Assay

The CRP concentrations in mouse sera were measured using a Mouse C-Reactive Protein ELISA Kit according to the protocol provided by the manufacturer. The quantity of CRP was interpolated from a standard curve.

### 2.9. Hematoxylin-Eosin (HE) Staining

Liver, lung, and kidney tissues were harvested on days 3 and 14 postinfection and stored in 4% paraformaldehyde. Pathological injury and inflammation in the HE-stained samples were observed and recorded.

### 2.10. Statistical Analysis

Data represent the average ± SD of each treatment group. The significance of the differences of survival rates between groups was analyzed using a log-rank (Mantel-Cox) test. A Student unpaired *t-*test was used to determine the significance of the difference between two independent datasets, two-way ANOVA was used to determine the significance of the difference between two groups, and *P* < 0.05 represents a significant difference.

## 3. Results

### 3.1. Effects of rhT*β*4 on Mice Infected with MHV-A59

**Figure 2:**
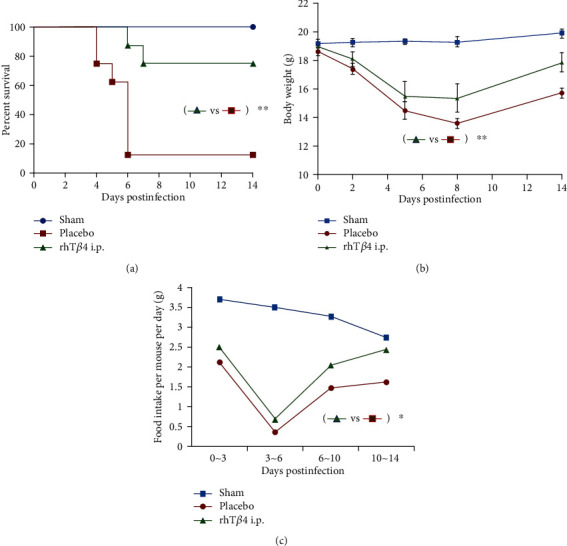
Protection of rhT*β*4 i.p in BALB/c mice by infection of MHV-A59. (a) The survival rate of mice in group sham, placebo, and rhT*β*4 i.p (*n* = 8 per group). Log-rank (Mantel-Cox) test was used to analyze the statistical difference between groups. ^∗∗^*P* < 0.01. (b) Change of body weights was monitored at 2, 5, 8, and 14 d.p.i. Two-way ANOVA was used to compare two group data. ^∗∗^*P* < 0.01. (c) Food intake of the survived mice in each group was calculated during 0 ~ 3, 3 ~ 6, 6 ~ 10, and 10~14 d.p.i. Two-way ANOVA was used to compared two group data. ^∗^*P* < 0.05.

Mice in the placebo group began to show typical signs of illness from day 2 postinfection, including difficulty walking, hunched posture, and erect and piled up hair. These mice began to die from day 4 postinfection, while mice in the rhT*β*4 group began to die on day 6 postinfection. The survival rates of mice in the placebo and rhT*β*4 groups were 12.5% and 75%, respectively (*P* < 0.01) (Figure 2(a)).

Mice in the placebo and rhT*β*4 groups lost appetite, and their body weights started decreasing on day 2 postinfection, compared with those of the mice in the sham group (Figure 2(b)). The weight loss of mice in the rhT*β*4 group was lower compared with that of the placebo group (*P* < 0.05) and gradually increased starting from day 8 postinfection. Food consumption of mice in the placebo and the rhT*β*4 groups significantly decreased after challenge. The lowest food consumption occurred from days 3 to 6 postinfection and then gradually increased (Figure 2(c)).

### 3.2. Anti-MHV Antibody Concentrations

**Figure 3:**
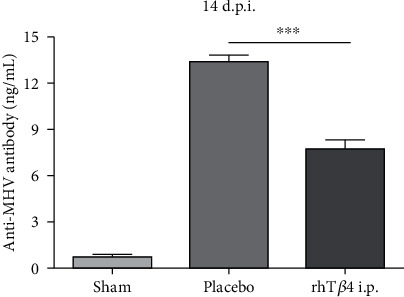
Anti-MHV antibody concentrations in serum at 14 d.p.i. The MHV antibodies in mice serum of group sham (*n* = 13), placebo (*n* = 2), AND rhT*β*4 (*n* = 11) were quantified by ELISA. The unpaired *t-*test was used to compare two group data. ^∗∗∗^*P* < 0.001.

MHV specific antibodies were detected in the sera of survivors on day 14 postinfection. MHV-A59 challenge elicited titers of specific antibodies in the placebo and rhT*β*4 groups. The anti-MHV antibody concentrations in group rhT*β*4 were significantly lower compared with those of the placebo group (*P* < 0.001) (Figure 3).

### 3.3. Viral RNA Copy Numbers in Tissues

**Figure 4:**
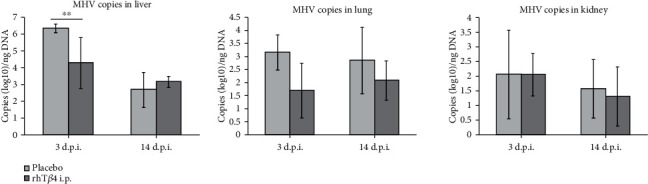
The virus RNA copies of MHV-A59 in the liver, lung, and kidney of mice. The MHV RNA copies in mice tissues of group placebo and rhT*β*4 were quantified real-time qPCR. The unpaired *t-*test was used to compare two group data. ^∗∗^*P* < 0.01.

The number of MHV-A59 RNA copies in the liver wasthe highest, followed by the lung and kidney. On day 3 postinfection, the viral RNA copies of liver tissues in the rhT*β*4 group were significantly lower compared with those of the placebo group (*P* < 0.01), and there was no significant difference between viral RNA copy numbers in the tissues of these group on day 14 after challenge. Further, these values did not significantly differ between the rhT*β*4 placebo groups (Figure 4).

### 3.4. Cytokine and CRP Levels

**Figure 5:**
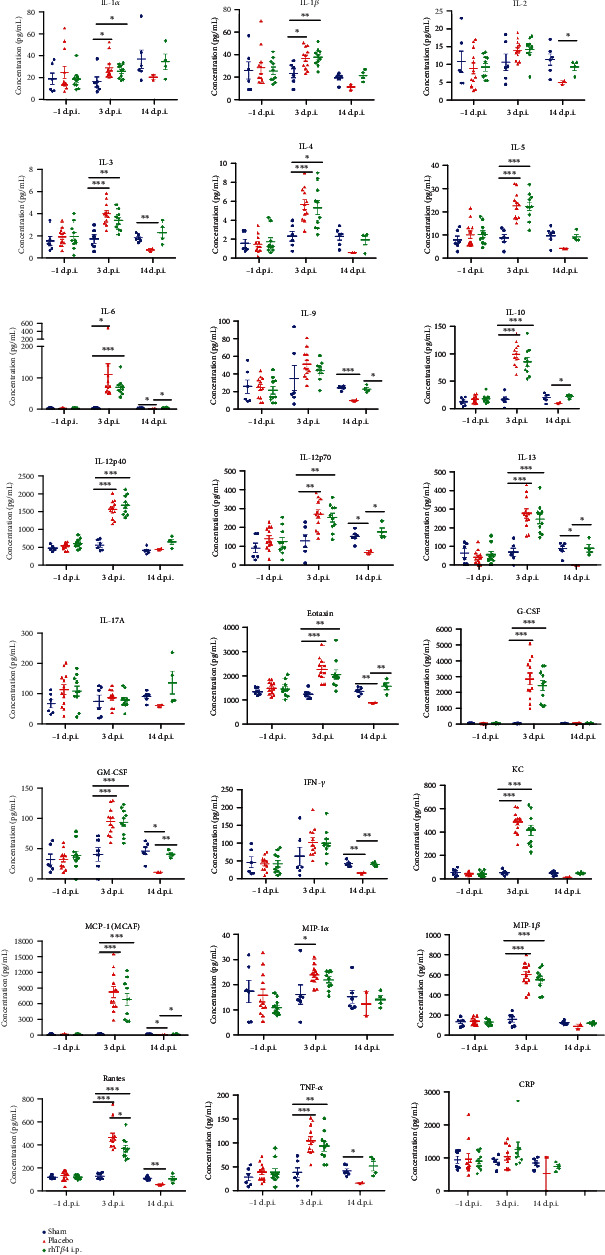
The cytokines and CRP concentrations in the mice serum. Cytokines in mouse sera were detected using a Bio-Plex Pro Mouse Cytokine 23-plex Assay Kit. The CRP concentrations in mouse sera were measured using a Mouse C-Reactive Protein ELISA Kit. The unpaired *t*-test was used to compare two group data. ^∗^*P* < 0.05, ^∗∗^*P* < 0.01, and ^∗∗∗^*P* < 0.001.

As shown in Table 1, on day 3 postinfection, the concentrations of 18 of 23 cytokines in the sera of both the placebo and rhT*β*4 groups were significantly increased vs normal values (*P* < 0.05). The levels of the four cytokines were not significantly different. On day 14 postinfection, the levels of the 23 cytokines in the rhT*β*4 group returned to normal, while the concentrations of 11 cytokines in the placebo group were significantly lower compared with normal values (*P* < 0.05). There was no significant change in the CRP concentration of each group during the experiment (Figure 5). These results indicate that MHV-A59 significantly inhibited the immune response of mice in the late stage of virus infection and during convalescence. However, rhT*β*4 effectively balanced the levels of proinflammatory and anti-inflammatory cytokines to maintain physiological immune homeostasis.

### 3.5. Protection from Tissue Injury

**Figure 6:**
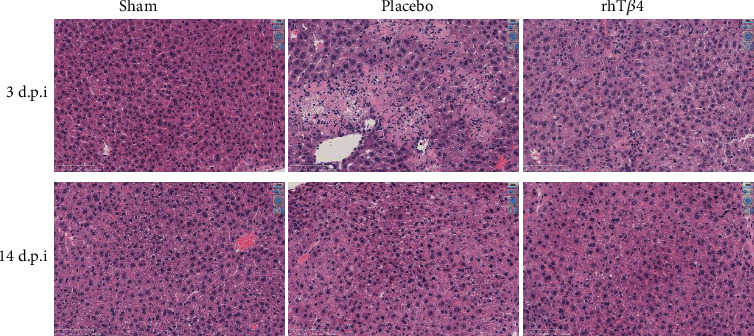
Pathological section of mice livers. Liver tissues were removed from the mice at 3 d.p.i. or 14 d.p.i., fixed with 4% paraformaldehyde for examination. The samples were dehydrated, embedded, and sliced for HE staining, and the pathologies of the livers were evaluated under microscope (Nikon Ci-S, Japan). The magnification of the pictures shown is ×40.

There was no significant injury to the lungs or kidneys of the placebo and rhT*β*4 groups. In contrast, MHV-A59 caused severe liver injury. For example, day 3 postinfection of mice in the placebo group caused hepatocyte necrosis, watery degeneration, inflammatory cell infiltration (including granulocytes and mononuclear cells), and bleeding in some necrotic areas of the liver. The rhT*β*4 group exhibited hepatocyte necrosis, watery degeneration, and minimal infiltration by inflammatory cells of the liver. Compared with mice in the placebo group, the rate and degrees of inflammatory cell infiltration of mice in the rhT*β*4 group decreased by day 14 postinfection, and hepatocyte necrosis was repaired in mice in the placebo group; although, there was watery degeneration and inflammatory cell infiltration (the number of Kupffer cells increased) and minimal hepatocyte regeneration. The rhT*β*4 group exhibited hepatocyte regeneration (binucleate and macronuclear regenerated hepatocytes). Compared with the placebo group, the livers of the rhT*β*4 group underwent injury repair, and hepatocyte proliferation was detected (Figure 6).

## 4. Discussion

Here, we show that MHV can be used as a model to screen for drugs that inhibit CoV infection under A-BLS2 containment. In contrast, studies of CoVs that are pathogenic for humans such as CoV-2 require A-BSL3 containment and the use of transgenic mice that express the CoV-2 receptor, human angiotensin-converting enzyme 2 [36].

The anti-inflammatory activities of T*β*4 suggested to us that it may serve to treat coronavirus infections of humans. Further, Han et al. found an association between reductions in serum levels T*β*4 and the severity of liver failure caused by chronic HBV infection [37], and Shahabuddin et al. found that chronic infection with HIV-1 reduces the level of T*β*4 in infected cells [38].

Here, we show that rhT*β*4 increased the survival rate of mice infected with MHV-A59, significantly reduced viral titers in the liver during the early stage of infection, protected the liver from severe injury, and promoted hepatocyte regeneration and tissue repair. There was no significant difference in the numbers of virus RNA copies of tissues between the placebo and rhT*β*4 groups during the later stage of infection. However, the anti-MHV-specific antibody titers of the rhT*β*4 group were significantly lower compared with those of the placebo group 14 days postinfection. This may be explained by the discordance between viral RNA copy numbers and the number of infectious viruses.

T helper cells (Th) play an important role in regulating the immune system. Th1 cells inhibit virus infection leading to inflammation or delayed type hypersensitivity through the secretion of cytokines such as IL-1, IFN-*γ*, and TNF-*β*. Th2 cells produce numerous cytokines (e.g., IL-4, IL-5, IL-6, IL-10, and IL-13) that induce B cells to mount a humoral immune response [39]. Th cells produce proinflammatory (e.g., TNF - *α*, IL-2, IL-6, IL-8, IL-12, IL-17, IL-23, IFN *γ*, and MIF) and anti-inflammatory cytokines (e.g., IL-4, IL-13, IL-10, TGF - *β*, and IL-1R *α*). Under normal physiological conditions, Th1 and Th2 cells maintain a balanced state of mutual inhibition and proliferation. The dynamic balance of proinflammatory and anti-inflammatory cytokines determines the development and outcome of inflammation. An imbalance in cytokine activities leads to pathology. Three days after MHV-A59 infection, the concentrations of anti-inflammatory and proinflammatory cytokines significantly increased, and all the physical characteristics of the mice, histopathology, cytokine assays suggested that the mice infected with MHV-A59 were in the “cytokine storm” state. The mice in the placebo group began to recover 14 days postinfection, as indicated by increased body weight and food consumption. However, the levels of 11 cytokines in the placebo group were significantly lower compared with their normal values, indicating that the virus significantly inhibited the activities of Th1 and Th2 cells to suppress the antiviral immune response. However, the level of each of the 23 cytokines produced by rhT*β*4-treated mice returned to normal 14 days after challenge. These results show that rhT*β*4 effectively balanced the activities of Th1 and Th2 cells as well as the concentrations of proinflammatory and anti-inflammatory cytokines to restore normal immune homeostasis.

Only two survived mice data were detected in the placebo group at 14 d.p.i., which is a small shortcoming of this study. Although we had tried different challenge doses of MHV-A59, no more mice in the placebo group could survive under the condition that the survival rate was statistically different, unless the sample size was particularly large. We will further increase the number of mice to acquire more data in the next study.

## 5. Conclusion

MHV-A59 can be used as a model virus to study CoVs under conditions of A-BSL2 containment. As proof of principle, we show here that although rhT*β*4 was not virucidal, it protected mice from virus challenge by balancing the immune response, promoting the regeneration of damaged cells, and repairing tissue damage, leading to rapid mitigation of the pathological consequences of CoV infection.

## Figures and Tables

**Table 1:** Comparison of cytokine levels in each group.

Days post infection	Compared to group sham	Group	
		Placebo	*rh*T*β*4
3 d.p.i.	Significantly higher	IL-1*α*,IL-1*β*, IL-3, IL-4, IL-5, IL-6, IL-10, IL-12p40, IL-12p70, IL-13, Eotaxin, G-CSF, GM-CSF, KC, MCP-1(MCAF), MIP-1*α*, MIP-1*β*, RANTES, TNF-*α*	IL-1*α*, IL-1*β*, IL-3, IL-4, IL-5, IL-6, IL-10, IL-12p40, IL-12p70, IL-13, Eotaxin, G-CSF, GM-CSF, KC, MCP-1(MCAF), MIP-*β*, RANTES, TNF-*α*
	Significantly lower	/	/
	No significantly different	IL-2, IL-9, IL-17A, IFN-*γ*	IL-2, IL-9, IL-17A, IFN-*γ*, MIP-1*α*
14 d.p.i.	Significantly higher	/	/
	Significantly lower	IL-3, IL-6, IL-9, IL-12p70, IL-13, Eotaxin, GM-CFS, IFN-*γ*, MCP-1(MCAF), RANTES, TNF-*α*	/
	No significantly different	IL-1*α*, IL-1*β*, IL-2, IL-4, IL-5, IL-10, IL-12p40, IL =17A, G-CSF, KC, MIP-1*α*, MIP-1*β*	IL-1*α*, IL-1*β*, IL-2, IL-3, IL-4, IL-5, IL-6, IL-9, IL-10, IL-12p40, IL-12p70, IL-13, IL =17A, Eotaxin, G-CSF, GM-CSF, IFN-*γ*, KC, MCP-1(MCAF), MIP-1*α*, MIP-1*β*, RANTES, TNF-*α*

## Data Availability

The data used to support the findings of this study are available from the corresponding author upon request.

## Additional Points

*Highlights*. This was the first report of recombinant human thymosin beta-4 (rhT*β*4) in the treatment of mouse coronavirus infection, which showed that rhT*β*4 significantly increased the survival rate of mice infected with MHV-A59 through inhibiting virus replication, balancing the host's immune response, alleviating pathological damage, and promoting repair of the liver. These results will serve as the basis for further application of rhT*β*4 to the treatment of human CoV diseases such as COVID-19.
